# Book Review

**Published:** 1917-03

**Authors:** 


### BOOK REVIEW

PRACTICAL URANALSIS. By B. G. R. Williams, M. D., director of Wabash Valley Research Laboratory, author of "Laboratory Methods," etc. One hundred and forty-two pages, illustrated. Limp cloth. The C. V. Mosby Company St. Louis, Mo. 1916.

Urinalysis should be regarded as a necessary part of every diagnostic examination. To simplify this subject and to give the essential details briefly Williams has written this concise little book which meets well the intentions of the author. Examinations for the minor products of erroneous metabolism and excretion are appropriately emphasized as being of importance in recognizing certain disturbed functions early in diseases when remedial measures may yet avail.

---

THE NEWER METHODS OF BLOOD AND URINE CHEMISTRY. By R. B. H. Gradwohl, M. D., Director of the Pasteur Institute, of St. Louis and the Gradwohl Biological Laboratories, St. Louis, and A. J. Blaivas, Assistant in the same; Sometime Technician in Pathological Chemical Laboratories, New York Post-Graduate Medical School and Hospital; and Former Assistant, Chemical Laboratory, St. Luke's Hospital, New York City. With sixty-five illustrations and four plates. The C. V. Mosby Company, St. Louis, Mo.

The writers have collected much valuable information dealing with the modern conception of this subject. Fortunately for the practitioner and student only one method is given for each test, for the manifest reason that the person needing the book is less able to judge the value of the tests than are the men who write the book. The book in consequence is more compact and clear.

